# Analysis of clinical efficacy after PKP in patients of different genders

**DOI:** 10.1097/MD.0000000000031785

**Published:** 2022-11-11

**Authors:** Bo Yang, Yangxue Zhao, Yu Zhao

**Affiliations:** a Xi’an Medical University, Xi’an, China; b Department of Orthopaedics, the Ninth Hospital of Xi’an, Xi’an, China.

**Keywords:** gender, osteoporotic vertebral compression fractures, percutaneous kyphoplasty, recurrent vertebral fracture

## Abstract

To investigate the clinical efficacy of Percutaneous Kyphoplasty (PKP) in male and female patients.

**Method:**

The clinical data of patients treated with PKP in our hospital from January 2018 to October 2020 were analyzed retrospectively. These patients were divided into female group and male group according to gender differences. The visual analogue scale (VAS) and activity function score (LAS) were recorded before operation, immediately after operation and 1 year after operation, and postoperative complications such as cement leakage and recurrent vertebral fracture were recorded. The above observation indexes between the 2 groups were statistically compared.

**Results:**

A total of 171 patients (118 in female group and 53 in male group) were included. There was no other difference between the 2 groups except that thoracic vertebral fracture was more common in women (*P* < .05). The VAS of female group and male group were (7.14 ± 0.61) and (7.11 ± 0.51) before operation, (3.05 ± 0.66) and (2.89 ± 0.64) after operation, 1 year after operation (1.10 ± 0.50) and (1.02 ± 0.37). There was no difference in each period between the 2 groups (*P* > .05). But there was a significant decrease after operation, which was recognized between the 2 groups (*P* < .05); The activity scores of female group and male group were (3.08 ± 0.48) and (3.07 ± 0.43) before operation, (1.86 ± 0.42) and (1.85 ± 0.50) after operation, 1 year after operation (1.01 ± 0.92) and (1.02 ± 0.14). There was no difference in each period between the 2 groups (*P* > .05). But there was a significant decrease after operation, which was recognized between the 2 groups (*P* < .05). Postoperative cement leakage was revealed in 21 cases (12.28%), 16 cases (13.56%) in female group and 5 cases (9.43%) in male group, which was no significant difference between the 2 groups. During the 1-year follow-up, postoperative recurrent vertebral fracture was revealed in 4 cases (2.34%), 2 cases (1.69%) in the female group and 2 case (3.77%) in the male group, which was also no significant difference between the 2 groups.

**Conclusion:**

Patients treated with PKP can immediately get pain relief and activity function improvement. PKP is a safe and effective minimally invasive surgery for both female and male patients.

## 1. Introduction

Osteoporosis, known as the silent killer, has swept the global elderly population. According to statistics, by the middle of this century, more than 200 million people in the world will suffer from osteoporosis due to bone mineral loss.^[1]^ China, the largest developing country, has a large population base and a high proportion of the elderly population. The proportion of osteoporosis is also great. It has been reported that nearly 10% of the patients with osteoporosis in magic city Shanghai, have undoubtedly brought great challenges to the construction of a healthy China.^[2]^

Unfortunately, bending and rolling over in everyday life can be a disaster for osteoporosis patients.^[3,4]^ Due to the high frequency of spine movement and rapid bone loss, fractures caused by osteoporosis are mostly located in the spine. Nowadays, minimally invasive interventional surgery, Percutaneous Vertebral Augmentation (PVA) has become the preferred treatment for patients with Osteoporotic Vertebral Compression Fractures (OVCFs). This technique makes the patient’s pain relieve quickly and the activity function improves significantly, so it has been unanimously praised by both doctors and patients.^[5–9]^

Although the advantages of PVA are well known, the safety and effectiveness of PVA for patients of different genders need to be clarified. The author consulted the literature and found that there were few comparative studies on the effect of Percutaneous Kyphoplasty (PKP) between patients of different sexes. If patients of different genders can be included in the applicable criteria for vertebral augmentation, almost all patients can enjoy the benefits of minimally invasive surgery. Therefore, it is necessary to analyze the results of PKP in patients of different genders. Hence, this paper retrospectively analyzed the clinical efficacy and safety of patients of different genders who received PKP treatment in our hospital from January 2018 to October 2020.

## 2. Materials and methods

### 2.1. Study target

The medical records of patients with OVCFs who received PKP from January 2018 to October 2020 in Ninth Hospital of Xi’an were reviewed, and 198 complete medical records were obtained. Among them, there were 61 male patients, 137 female patients. The subjects were divided into female group and male group according to gender differences. Retrospective studies were performed according to the following inclusion and exclusion criteria. Inclusion criteria: magnetic resonance imaging-proven thoracolumbar OVCFs. No vertebral body has been augmented with cement in the past. The patient is conscious and free of Alzheimer’s disease and other diseases, and can cooperate with the research. The posterior wall of the vertebral body is intact without damage. Regular anti-osteoporosis treatment after operation. Exclusion criteria: Compression fracture of vertebral body secondary to tumor, Symptoms of nerve compression, Continuous follow-up less than 12 months.

### 2.2. Technical method

The patient was placed in the prone position with the abdomen suspended and the body position was comfortable. The operative vertebra and its pedicle projection were determined with the help of C-arm machine. Routine sterilization and drape, take the left side of the pedicle of the operated vertebra at 9 o’clock and the right side at 3 o’clock and gradually enter into local infiltration anesthesia. Insert 11-gauge puncture needle into the anesthesia channel. The position and depth of the puncture needle are appropriate under the anterolateral C-arm fluoroscopy (the puncture needle reaches the inner edge of the pedicle on the frontal film, and the puncture needle reaches the posterior edge of the operated vertebra on the lateral film). Then the puncture needle was removed and the guide wire was inserted so that the tip was located in the middle of the vertebral body on the anteroposterior radiograph, and the anterior and middle 1/3 of the vertebral body on the lateral radiograph. The working cannula and bone drill were placed, and the original guide wire entry trajectory was expanded and compacted so that the kyphoplasty balloon could be placed smoothly. The bone drill was then pulled out and the kyphoplasty balloon was inserted. The balloon was pressurized to 180 mm Hg, and C-arm fluoroscopy was used to see that the upper and lower endplates of the vertebral body were intact. Pull out the balloon and inject cement into the vertebra during the wire drawing. Inject 1/6 tube of cement every time, and conduct fluoroscopy once. See that the cement is well filled, and then complete the cement injection. Once the trend of cement leakage to the posterior edge of the vertebral body is found during the operation, the injection of cement shall be stopped. After completing the cement injection, disinfect and bandage the wound, assist the patient to turn over when the cement sample on the sterile table has no fever reaction, check the patient’s lower limbs feel and move well, and then end the operation.

### 2.3. Post-operative treatment plan

After surgery, patients were kept in bed for 1 day, and on the second day, they got out of bed with a brace and reviewed the imaging data. If there was no abnormality, they could be discharged from the hospital. Zoledronate was given anti-osteoporosis treatment during hospitalization, and continued regular oral calcitriol, calcium carbonate D3 and other symptomatic anti-osteoporosis treatments after discharge.

### 2.4. Observation parameters

The changes of pain in patients were recorded by the commonly used visual analogue scale (VAS). A score of 0 means no pain, and a score of 10 means extreme pain. A score of 0 to 10 means that the pain symptoms are progressively worsened. The VAS before operation, immediately after operation, and 1 year after operation were recorded in the patient’s conscious state. Changes in patient activity and function were assessed using the LAS score in previous studies.^[10]^ The score ranges from 1 to 4 points, with 1 point representing unrestricted performance in daily activities, which is not significantly different from ordinary people. A score of 2 means that the patient has difficulty walking and can only complete part of the walking goal. A score of 3 means unable to walk alone, needs to be assisted by a wheelchair or can only sit. A score of 4 means that the patient is afraid of walking, severely limited in daily activities, and can only stay in bed. More importantly, the intraoperative and postoperative complications of the patients were also fully recorded. The specific point is to record the patients’ intraoperative C-arm fluoroscopy and postoperative CT examination of cement leakage. The patients were followed up for at least 12 consecutive months after the operation. During the follow-up period, once the patient complained of low back pain, magnetic resonance imaging of the thoracic and lumbar spine was performed to determine whether there was any recurrence of vertebral fractures.

### 2.5. Statistical analysis

Statistical Packages for Social Sciences18.0 software (IBM) was used for statistical analysis of data. The continuous data is first judged whether it is normally distributed. If it conforms to the normal distribution and the variance is homogeneous, it is expressed as (x ± s), and the continuous data between the 2 groups is compared by the t test, and the chi-square test is used for the categorical data between the 2 groups. Comparison. The test level was 0.05 on both sides, with *P* < .05 as the difference, which was statistically significant.

## 3. Results

### 3.1. Patient characteristics

After careful correction of patient information, 27 patients were excluded (8 patients have received PKP in the past, 17 patients could not complete the follow-up, 2 patients with secondary vertebral fractures of tumors), so 171 patients who met the criteria were finally included. Among them, there were 118 cases in the female group and 53 cases in the male group. The average age of patients in the female group was 74.64 ± 8.79 years (57–95), and the average age in the male group was 76.68 ± 8.89 years (57–95), there was no significant difference between the groups. In addition, there were no differences in body mass index, operation time, intraoperative blood loss, and cement injection volume between the 2 groups. However, the comparison between the 2 groups of vertebral body distribution position found that the incidence of thoracic vertebral body was more common in women (*P* < .05) (Table 1).

**Table 1:** Comparison of general data between the 2 groups.

	Female (n = 118)	Male (n = 53)	Total (n = 171)	*P* value
Age	74.64 ± 8.79	76.68 ± 8.89	75.27 ± 8.83	.162
BMI	22.54 ± 2.96	22.84 ± 2.57	22.63 ± 2.84	.528
Thoracic vertebra	64	18	72	.019
Lumbar vertebra	72	40	112	.150
Cement volume (mL)	5.50 ± 1.63	5.71 ± 1.63	5.57 ± 1.72	.475
Operation time (min)	51.19 ± 11.94	48.87 ± 8.36	50.47 ± 10.98	.203
Intraoperative blood loss (mL)	17.84 ± 7.72	18.68 ± 8.56	18.10 ± 7.79	.526

The comparison between the 2 groups of vertebral body distribution position found that the incidence of thoracic vertebral body was more common in women (*P* < .05).

BMI = body mass index.

### 3.2. Clinical outcomes and complications

**Figure 1.**
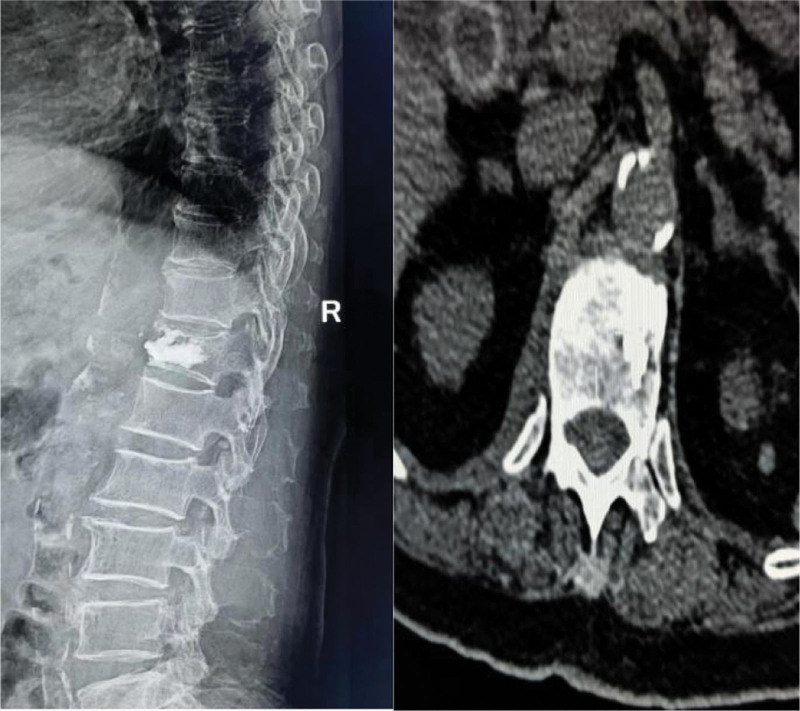
In a 73-year-old woman, we performed PKP for her T12 fracture. Postoperative follow-up DR and CT showed leakage of paravertebral bone cement. However, the patient did not have any symptoms. PKP = percutaneous kyphoplasty.

**Figure 2.**
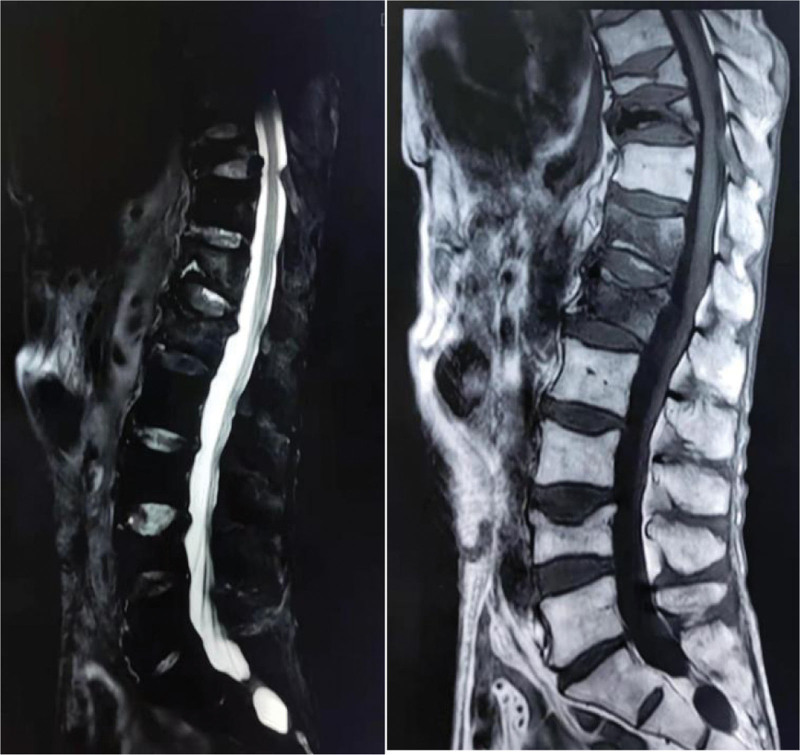
A 85 year old man was treated with PKP for T11 and L 1, but his back pain recurred 1 month after operation. MRI showed the fracture of T12. MRI = magnetic resonance imaging, PKP = percutaneous kyphoplasty.

All 171 patients successfully completed PKP. The VAS of the female group before operation, Immediately after operation and 1 year after operation were (7.14 ± 0.61), (3.05 ± 0.66), (1.10 ± 0.50), respectively; the VAS of the male group before operation, Immediately after operation and 1 year after operation were (7.11 ± 0.51), (2.89 ± 0.64), and (1.02 ± 0.37). Immediately after surgery and 1 year after surgery, the VAS of the 2 groups were significantly lower than those before surgery, and there was no difference between the groups at each time period (Table 2). The female group’s LAS before operation, Immediately after operation and 1 year after operation were (3.08 ± 0.48), (1.86 ± 0.42), (1.01 ± 0.92), respectively; the male group’s LAS before operation, immediately after operation, 1 year after operation were (3.07 ± 0.43), (1.85 ± 0.50), and (1.02 ± 0.14), the LAS activity scores of the 2 groups of patients immediately after operation and 1 year after operation were also significantly lower than those before operation, and at each time period there was no difference between groups (Table 3). This shows that the improvement of pain and activity function in patients after PKP is clear and beyond doubt. A total of 21 cases (12.28%) of cement leakage occurred during the operation (a typical example is shown in Fig. 1), 16 cases (13.56%) in the female group and 5 cases (9.43%) in the male group. There was no significant difference between the 2 groups (Table 4). During the postoperative follow-up period, 4 cases (2.34%) of recurrent fractures occurred (a typical example is shown in Fig. 2), 2 cases (1.69%) in the female group and 2 cases (3.77%) in the male group, and there was no significant difference between the groups (Table 5).

**Table 2:** VAS of 2 groups.

	Pre-VAS	Post-VAS	VAS (1 year after operation)	*P* value (compared with preoperative)
Female (n = 118)	7.14 ± 0.61	3.05 ± 0.66	1.10 ± 0.50	<.001
Male (n = 53)	7.11 ± 0.51	2.89 ± 0.64	1.02 ± 0.37	<.001
*P* value (comparison between 2 groups)	0.816	0.133	0.225	

Immediately after operation and 1 year after operation, the visual analogue scale (VAS) of the 2 groups were significantly lower than those before operation, and there was no difference between the groups at each time period.

**Table 3:** LAS of 2 groups.

	Pre-LAS	Post-LAS	LAS (1 year after operation)	*P* value (compared with preoperative)
Female (n = 118)	3.08 ± 0.48	1.86 ± 0.42	1.01 ± 0.92	<.001
Male (n = 53)	3.07 ± 0.48	1.85 ± 0.50	1.02 ± 0.0.14	<.001
*P* value (comparison between 2 groups)	0.905	0.926	0.561	

The LAS activity scores of the 2 groups of patients immediately after operation and 1 year after operation were also significantly lower than those before operation, and at each time period there was no difference between groups.

**Table 4:** Complications of cement leakage.

	Leakage	Well	
Female (n = 118)	16	102	
Male (n = 53)	5	48	
Total	21	150	
*P* value			.615

A total of 21 cases (12.28%) of cement leakage occurred during the operation, 16 cases (13.56%) in the female group and 5 cases (9.43%) in the male group. There was no significant difference between the 2 groups.

**Table 5:** Complications of recurrent fractures.

	Fractures	Well	
Female (n = 118)	2	116	
Male (n = 53)	2	51	
Total	4	167	
*P* value			.589

During the postoperative follow-up period, 4 cases (2.34%) of recurrent fractures occurred, 2 cases (1.69%) in the female group and 2 cases (3.77%) in the male group, and there was no significant difference between the groups.

## 4. Discussion

Before puberty is the bone reserve period of the human body, during this period, bone synthesis is the main direction of bone metabolism. After puberty, hormone metabolism in the body changes, resulting in a gradual loss of bone mass. In old age, the bone reserve in the body is seriously insufficient, and a large amount of bone minerals is lost, resulting in changes in the microstructure such as trabecular bone. This evolution process makes most elderly people susceptible to osteoporosis.^[11]^ Osteoporosis can cause acute and chronic pain. Chronic pain is back pain and leg cramps caused by long-term bone loss. On this basis, low-energy trauma such as cough and turning over can cause vertebral fractures, causing acute pain in patients and aggravating the economic burden of the family.^[12,13]^ OVCFs, as one of the common osteoporotic fractures, has long caused orthopedic doctors to think about its diagnosis and treatment technology. The traditional reduction and screw rod internal fixation has insufficient holding force and is easy to loosen, and there are many basic cardiopulmonary diseases in elderly patients, with a high rate of surgical contraindications. Therefore, PVA has become the preferred treatment for OVCFs patients.^[14,15]^ In clinical operation, direct injection of cement into the injured vertebra has a high injection pressure and a high rate of cement leakage. Therefore, PKP with pre-balloon expansion and compaction to create a low-pressure cavity has become a more commonly used surgical procedure in clinical practice, so all patients in this study received PKP.^[16,17]^

When comparing the basic data of the 2 groups of patients, it was found that the female group received 64 PKP treatments for the thoracic vertebrae, while the male group only received 18 PKP treatments for the thoracic vertebrae, which was statistically significant. The author speculates that this is because male patients have more daily activities, causing fractures to occur more frequently in the lumbar spine, which has the greatest mobility in the spine, thereby reducing the incidence of thoracic fractures and making thoracic fractures significantly less than female patients.

Patients with OVCFs experience excruciating pain due to stimulation of the sinus vertebral nerves around the vertebral bodies. After the cement is injected into the injured vertebra, the heat generated by the polymerization reaction can instantly destroy the sinus and vertebral nerves, suddenly reduce the pain of the patient, and improve the patient’s mobility.^[18]^ In this study, the original pain of the 2 groups of patients after PKP was significantly reduced, and the VAS scores immediately after and 1 year after surgery were significantly different from those before surgery. Consistent with Baz et al^[19]^’s results of PKP for OVCFs, the average VAS recorded in the preoperative patients was 7.97, the postoperative drop was 2.21, and it was 1.98 at the last follow-up. It is also consistent with the result that the VAS score decreased from 7.3 before surgery to 3.3 at 24 hours after surgery in previous foreign studies.^[20]^ Before surgery, patients with OVCFs had to use wheelchairs to complete their daily activities due to pain, and were even unable to move and were absolutely bedridden. The preoperative LAS scores of the 2 groups were 3.08 ± 0.48 in the female group and 3.07 ± 0.43 in the male group. After the operation, the patient’s activity function improved significantly, and thy could walk alone to complete their daily life. Immediately after surgery and 1 year after surgery, the LAS scores were significantly lower than those before surgery, and there were statistically significant differences. This shows that patients can achieve daily activities without obvious obstacles after surgery, and avoid complications such as lower extremity venous thrombosis, hypostatic pneumonia, and urinary tract infection caused by prolonged lying.^[21]^

In the liquid phase, the cement is injected into the vertebral body through a push rod, and it can spread freely through the fracture space. However, once it enters the paravertebral vein and enters the main blood supply area such as the pulmonary artery, it can immediately cause the patient’s life to be in danger. In reviewing the literature, the author found that since Chen HL et al^[22]^ reported earlier on cement pulmonary embolism, there have been several studies that unfortunately had this disaster.^[23–26]^ Although cement leakage is extremely harmful, most of the leakages in the existing studies are asymptomatic. The author reviewed the literature and found that the cement leakage rate can be as high as 76%,^[27]^ which is much higher than the 12.28% in this study. At the same time, all 21 patients with cement leakage in this study were asymptomatic. Although the cement leakage rate in the female group was 13.56% higher than that in the male group, 9.43%, no statistical difference was found after comparison. There are many factors affecting cement leakage in research reports, such as bone integrity, cement viscosity, and cement injection volume.^[28–30]^ In this study, all patients who underwent PKP had no cortical damage to the vertebrae. The chief surgeon slowly injected the cement into the intended vertebra during the wire drawing period, and did not pursue a filling mode that fully contacts the upper and lower endplates. Therefore, the leakage rate of cement is low in this study.

As a common complication, recurrent vertebral fracture after PVA is less harmful than cement leakage, but it reduces the sense of benefit of minimally invasive surgery. It has also attracted the attention and research of doctors. In this study, there were 4 cases (2.34%), 2 cases (1.69%) in female group and 2 cases (3.77%) in male group during postoperative follow-up. There was no significant difference between the 2 groups. This indicates that there is no gender difference in recurrent vertebral fracture after operation. As early as 2002, Berlemann U et al^[31]^ studied the biomechanics of adjacent vertebra after PVA. They found that the normal vertebral body stress of adjacent segments increased after PVA, and the risk of fracture was significantly increased. In 2003,^[32]^ foreign physicians reported that 36 new vertebral fractures occurred in 177 patients who received PVA 2 years after surgery. Gradually, there are many studies on the recurrence of vertebral fractures after PVA. In previous reports, the recurrence rate of vertebral fractures after PVA can reach about 50%.^[33]^ In contrast, the incidence of recurrent vertebral fractures was 2.34% in this study, which was significantly lower than that reported in the literature. The author thinks that this is because we routinely chooses bilateral pedicle puncture and injection of cement. During the operation, the cement is distributed across the midline of the vertebral body, which can achieve uniform filling of the injured vertebra. In turn, the adjacent vertebra are evenly stressed and the rate of recurrent vertebral fractures is reduced. Some studies suggest that the main risk factors for recurrent vertebral fracture after operation are the number of fractured vertebral bodies, the volume of cement injected, the leakage of cement intervertebral disc and the distribution of bone cement. Therefore, the authors suggest that PKP can avoid these risk factors, so as to reduce the rate of recurrent fracture and improve the sense of minimally invasive benefit of patients.^[34–36]^

However, some limitations still exist in our study. It is a retrospective study and is highly biased. Furthermore, the scale of the included cases was small and the follow-up period was short. Therefore, further prospective, large-sample, multicenter studies are needed.

## 5. Conclusion

Patients treated with PKP can immediately get pain relief and activity function improvement. PKP is a safe and effective minimally invasive surgery for both female and male patients.

## Author contribution

Bo Yang conceived the research design, Bo Yang collected data and papered the manuscript, Yu Zhao revised this article, Yu Zhao is responsible for this article.

**Conceptualization:** Yu Zhao.

**Data curation:** Bo Yang.

**Formal analysis:** Bo Yang.

**Investigation:** Bo Yang.

**Methodology:** Bo Yang.

**Resources:** Bo Yang.

**Software:** Bo Yang.

**Supervision:** Yu Zhao.

**Validation:** Bo Yang.

**Visualization:** Bo Yang, Yangxue Zhao, Yu Zhao.

**Writing – original draft:** Bo Yang.

**Writing – review & editing:** Bo Yang.
